# Neurosurgical treatment and outcome patterns in patients with meningioma in South Moravian region—a population-based study

**DOI:** 10.1007/s10072-023-07244-3

**Published:** 2023-12-27

**Authors:** Miloš Duba, Andrej Mrlian, Ivana Rošková, Martin Smrčka, Pavel Fadrus, Jaroslav Duba, Ondřej Hrdý, Dagmar Al Tukmachi

**Affiliations:** 1 Department of Neurosurgery, University Hospital Brno, Brno, Czech Republic; 2 Faculty of Medicine, Masaryk University, Brno, Czech Republic; 3 Department of Anaesthesia, Resuscitation and Intensive Medicine, University Hospital Brno, Brno, Czech Republic; 4 Central European Institute of Technology, Brno, Czech Republic

**Keywords:** Meningioma, Diagnostics, Treatment, Follow-up

## Abstract

**Introduction:**

Meningiomas are usually slow-growing tumours, constituting about one third of all primary intracranial tumours. They occur more frequently in women. Clinical manifestation of meningiomas depends on their location, tumour size and growth rate. In most cases, surgical treatment is the procedure of choice. The success of this treatment is, however, associated with the radicality of the resection. Radiotherapy represents an additional or alternative treatment modality. Gamma knife surgery is another notable treatment method, especially in small and/or slow-growing tumours in eloquent areas or in elderly patients.

**Material and methods:**

Authors describe their experience with the diagnosis, treatment and outcome of the patients with meningioma (*n* = 857). Furthermore, they also assess the postoperative morbidity/mortality and recurrence rate.

**Results and conclusions:**

In view of the benign histology of meningiomas, the success of the treatment largely depends (besides the tumour grading) on the radicality of the resection. The emphasis is also put on appropriate follow-up of the patients. In certain patients, the watch and wait strategy should be also considered as a suitable treatment method.

## Introduction

Meningiomas are the most common type of primary brain tumour, accounting for approximately one third of all brain tumours [1]. They are usually slow-growing, often calcified tumours that arise from the arachnoideal cells. Their behaviour varies from explicitly benign (WHO grade I), through a group of tumours with aggressive and recurrent growth, to several forms of atypical or anaplastic meningiomas (WHO grade II, III) [1, 2]. The management of meningiomas is a critical issue, as early detection and prompt treatment are essential for ensuring the best possible outcomes for patients. In Czechia, the incidence of meningiomas is reported to range between 2 and 6 per 100,000 persons per year. Many meningiomas are clinically silent, which may cause differences in the reported incidences between reports based on live patients and autopsies [3]. The maximum incidence is at the age of approx. 45 years; meningiomas are more common in women (2:1) [4]. However, some authors have reported a higher incidence of more malignant variants of meningiomas in men [5]. Between 1 and 4% of meningiomas occur in childhood, and about a quarter of them are associated with Recklinghausen’s disease. In such cases, multiple occurrences are very common. Multiple meningiomas can also occur as a consequence of radiotherapy for another intraaxial tumour. Surgical resection is the primary treatment modality, and it is often curative. The goal of the surgery is to remove the entire tumour while preserving neurological function. In many cases, this can be achieved with complete resection. Simpson grading system, despite being developed as soon as in 1950s [6], remains one of the most useful methods for describing the resection radicality and evaluating the risk of recurrence. Simpson 1 (hereinafter abbreviated as S1) indicates practically complete resection of the tumour including the attached dura mater. It was shown to be associated with 10% 10-year recurrence [7]. However, in some cases, the tumour may be located in a critical area of the brain and it is making complete resection difficult or impossible [8]. Simpson 2 (S2) indicates a complete resection of the tumour without dura mater, which is cauterized. Simpson (S3) indicates that the meningioma is not supposed to be removed completely due to the high risk of damage of several important structures such as a cavernous sinus or the confluence of sinuses. A small piece has to be left and it is suitable for adjuvant therapy. Simpson 4 (S4), which is associated with the highest 10-year recurrence rate (40%), indicates only partial removal of the tumour, which may, however, still be useful for reduction of intracranial pressure or improving the suitability for radiotherapy (gamma knife, Cyberknife) [9]. Simpson 5, i.e. biopsy, is at present, due to the great progress in imaging methods over the last 70 years, obsolete.

The Department of Neurosurgery at the University Hospital Brno is equipped with modern equipment and highly trained personnel experienced in meningioma management. The University Hospital has access to the latest imaging techniques, including magnetic resonance imaging (MRI), tractography and functional MRI. We also use state-of-the-art surgical techniques, including image-guided surgery, to ensure precise removal of the tumour while minimizing the risk of neurological damage [10]. In addition to (or, in some cases, instead of) surgical management, radiotherapy may be used to treat meningiomas that cannot be completely removed, those that are located in critical areas of the brain, or in tumours with a higher degree of malignancy. In the latter, radiotherapy is also used as adjuvant therapy. In cases with multiple meningiomas with unfavourable localization, radiosurgery should be considered or even preferred [11]. The cooperating hospital, Masaryk Oncological Centre Brno, is able to provide advanced radiation therapy techniques, including stereotactic radiosurgery as well as the intensity-modulated radiation therapy. Furthermore, multidisciplinary teams including neurosurgeons, radiation oncologists and medical oncologists collaborate to provide comprehensive care for meningioma patients and ensure that the patients receive the best possible treatment and management plan tailored to their specific needs. Meningiomas can be also treated with radiosurgery (Leksell’s gamma knife) [9]. This method is indicated especially in smaller, slow-growing, practically asymptomatic tumours located in the eloquent area (such as the chiasm, cavernous sinus and pontocerebellar angle), or in elderly patients, where open surgery would be associated with higher morbidity and mortality risk.

Overall survival rate depends on several factors and, therefore, data for benign and malignant forms should be evaluated separately. Five-year survival in benign forms varies between 70 and 90%. In malignant variants, the 5-year survival is just over 50%. In general, patients with benign forms, women, tumours less than 2.5 cm and patients who underwent surgery without the necessity for adjuvant radiotherapy have better survival [5, 8, 11].

In addition to the above-described prognostic factors (radicality of surgery, WHO tumour classification, etc.), miRNA appears to be another potential promising prognostic tool. In the last 10 years, there has been significant interest in elucidating the role of micro-RNA (miRNA) in carcinogenesis. These small non-coding RNAs post-transcriptionally regulate the expression of the target mRNA [12]. The role of miRNAs in meningiomas has been also investigated in association with the biological properties of these tumours [12, 13]. It is assumed that certain miRNAs could act as key regulators in meningiomas and, regardless of the classic histopathological grading, could provide a more accurate prediction of the tumour behaviour. Deregulation of miRNA levels was observed both in connection with meningiomas and with radioresistance of these tumours. For this reason, we can hope that in the near future, miRNA analysis could serve as a promising tool for more accurate prediction of the risk of meningioma progression and/or recurrence [12, 13].

The clinical manifestation of meningiomas depends mostly on the location and the size of the tumour. However, there are several unspecific clinical signs that could focus our attention to other diseases. Therefore, the diagnostics usually starts with a CT scan, which can provide a very good idea of the diagnosis. It could show calcifications, extraaxial growth or displacement of midline structures, or significant post-contrast enhancement. However, gadolinium MRI (magnetic resonance imaging) remains the golden standard. The MRI scans typically show clear border between the meningioma and physiological tissue, which is not typical of malignant cerebral tumours and improves the reliability of diagnosis. In most cases, a layer of cerebrospinal fluid can be seen, which facilitates the surgical removal of the tumour. Angiography can be also helpful, because it informs us about the relationship of the tumour to the surrounding large vessels and describes the potential vascularization of the tumour. Angiography can also in some cases serve as a treatment method—it can be used for embolization of the nutritional arteries, which can reduce the blood loss during the surgery [14].

## Results

### Baseline characteristics

**Fig. 1:**
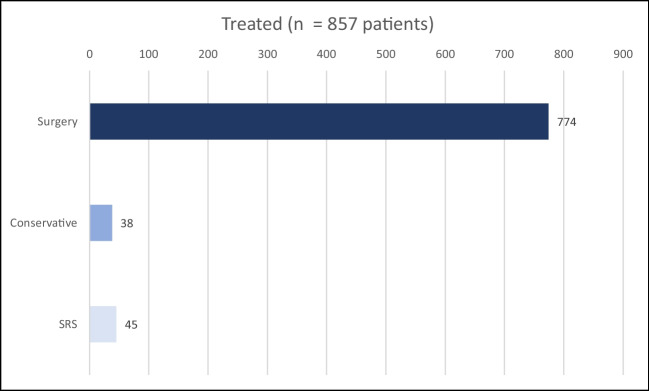
Baseline characteristics of the treatment modality of the whole group of patients (*N* = 857). SRS stereotactic radiosurgery

**Fig. 2:**
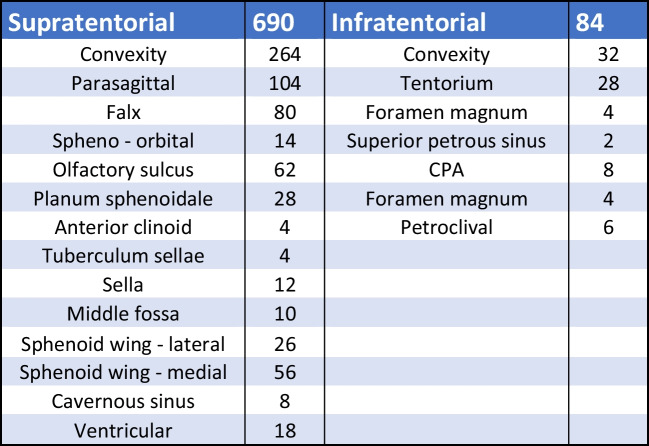
Anatomical location of surgically treated meningiomas. CPA cerebellopontine angle

**Fig. 3:**
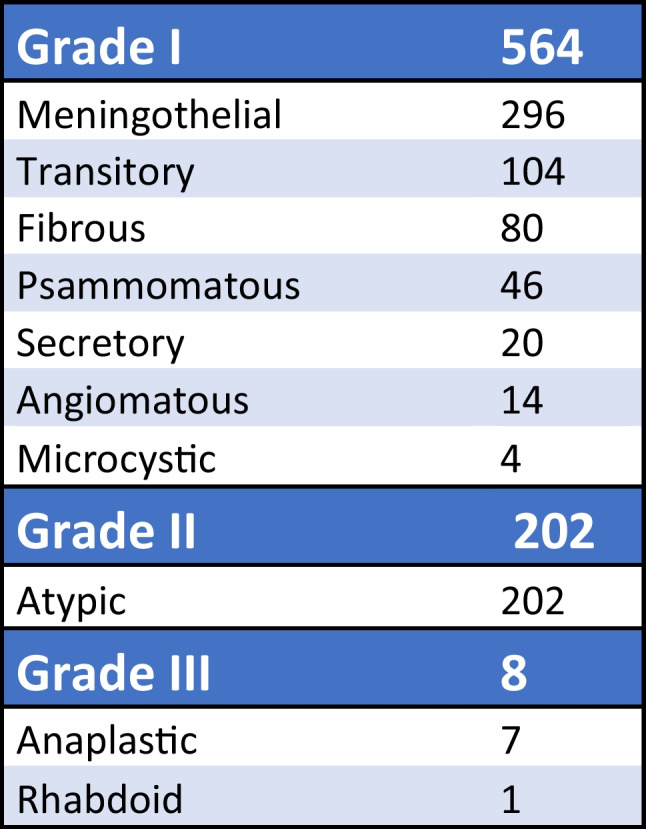
The division of surgically treated meningiomas according to the histological results

Between years 2005 and 2020, 857 meningioma patients were treated in our department. Male:female ratio of the group was 1:2.3. Seven hundred seventy-four patients underwent surgical procedure; furthermore, we left 38 patients for observation (watch and wait strategy) and 45 patients were treated only with stereotactic radiosurgery (Fig. 1). The age range of the whole group was 26–86 years; the median age was 61 years. We performed surgery in 218 men and 556 women. In 760 cases, the tumour was removed using craniotomy; 14 patients were operated using the transnasal approach. In terms of tumour localization, two groups of patients could be distinguished: 690 patients had a tumour in a supratentorial location and 84 patients suffered from an infratentorial location of the tumour (Fig. 2). In terms of the degree of malignancy, meningiomas can be divided into three subgroups (grading) according to the WHO classification [1]. Obviously, histological classification could be performed only in surgically treated patients (*n* = 774). Five hundred sixty-four of our patients had grade 1 meningioma, 202 patients had grade 2 meningioma, and 8 patients were treated for grade 3 meningioma. A more detailed histological analysis further classified these patients into more detailed subgroups (Fig. 3).

### Treatment results

**Fig. 4:**
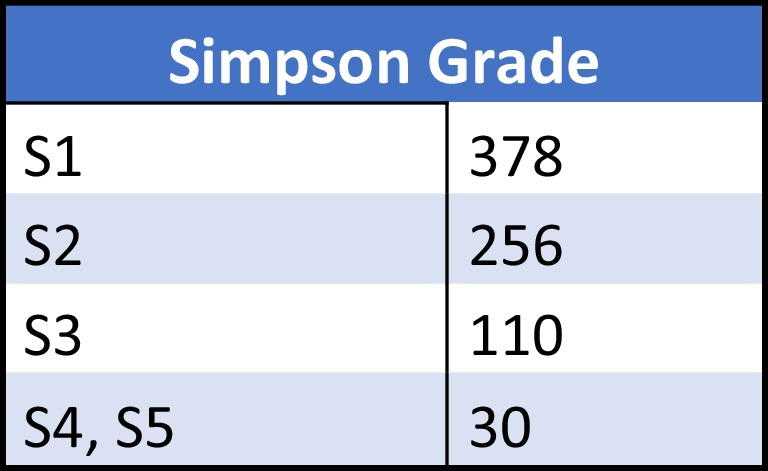
The division of surgically treated patients in accordance of radicality of resection classified after the Simpson grade (*n* = 774) [6]. Eighty-two percent of the patients underwent a complete resection with or without the resection of attached dura mater

The radicality of operations classified according to Simpson grade is documented in Fig. 3. Three hundred seventy-eight patients underwent radical resection including the excision of the affected dura mater or adjacent bones (S1); in 256 cases, a macroscopic total resection took place and the dura was treated with bipolar cauterization (S2); 110 patients had the tumour removed (near total resection) without resection of the dura (S3); and in 30 cases, the tumour was only partially removed—the radicality of the surgery ranged from biopsy to partial resection (S4, S5; Fig. 4).

Furthermore, we monitored the 30-day morbidity, mortality and surgical complications in the surgically treated patients. The mortality of the group within the first 30 days after the surgery was 0.005% (*n* = 4), two patients died from cardiac arrest and the other two deceased patients suffered a severe pulmonary embolism.

Development of brain oedema caused by the disruption of blood–brain barrier and empty space after the removed tumour was relatively common in our patient group. This complication was observed in 12.5% of the patients (*n* = 97), with severity ranging from minimal MR scan changes (T2 sequence) to significant midline shift requiring urgent surgery due to the deterioration of consciousness. Seventeen patients underwent decompressive craniectomy; the rest of the patients have been successfully treated with accelerated dosage of mannitol and corticosteroids.

Symptomatic postoperative hematoma was registered in 9.3% of the patients (*n* = 72), of which 55 patients required additional surgery. In 25 patients, the postoperative period newly developed a seizure. Sixteen patients required permanent antiepileptic pharmacotherapy; 9 patients had only a solitary partial or generalized seizure resolved by a single dose of benzodiazepines or phenytoin.

One hundred twenty-seven patients (16%) showed cerebrospinal fluid leakage. Of those, 3 patients suffered from nasal cerebrospinal fluid leak after a transnasal transsphenoidal surgery, of which one patient had to be revised and 2 were treated conservatively, using lumbar spinal drainage. In the remaining 124 patients, the leakage manifested as a subgaleal accumulation of cerebrospinal fluid due to a possible insufficient (not watertight) dura mater closure. As a first step, we used lumbar spinal drainage and maximal conservative treatment (anti-Trendelenburg position, fluid restriction, wound compression with an elastic bandage). Where the leak could not be resolved satisfactorily by conservative methods within 7 days, we proceeded with extraction of the lumbar spinal drainage to prevent of infectious complications and proceeded to surgical revision that was needed in 77 patients (10%). Most of those were patients after complete resection (S1) with convexity meningiomas located near the midline in the region of the vertex (*n* = 27) or above the posterior fossa (*n* = 22).

Twenty-nine patients (3%) developed a permanent neurological deficit within the first 30 days after the surgery. All of them were patients with a meningioma located in eloquent zones, such as the central motoric cortex, posterior fossa or planum sphenoidale. While the deficit in patients with tumour near the central motor cortex lied in hemiparesis, damage to one of the cranial nerves (olfactory, optic, oculomotor, trigeminal, facial, vestibulocochlear or lower cranial nerves) was observed in those with tumours in the posterior fossa or planum sphenoidale.

An infection occurred in 24 patients (3%) within the monitored 30-day period. All 24 patients required an additional surgery for abscess evacuation and bone flap removal. Of these patients, 11 had planum sphenoidale meningioma and during the original craniotomy, their frontal sinuses were opened. The remaining 15 patients represented a very diverse group with various reasons of infection development. The surgical revision included a lavage with Betadine solution or hydrogen peroxide, followed by intravenous administration of antibiotics, most often cefotaxime in combination with metronidazole. After 3 weeks, the long-term antibiotic treatment was converted to oral administration (altogether 3 months). After this period, a custom-made artificial bone flap was inserted in place of the removed own bone.

The patients were subsequently followed up with frequency depending on the tumour grade and radicality of resection (see below in “Discussion”). The average follow-up period of the whole group is 10 years; the maximum is 15 years so far. During the follow-up, 41 recurrences (5.3%) associated with a significant clinical deterioration and a correlating MR scan have occurred so far. Twenty-three recurrences occurred in patients with grade I meningioma (i.e. 4% of patients with grade I meningiomas). The primary surgery was radical (S1) in 15 patients from this group, the radicality S2 was achieved in 5 patients and in the remaining 3 patients, the original tumour was completely removed but dura mater could not have been treated (S3). In patients with grade 2 meningiomas, 32 recurrences were observed (15.8% recurrence rate). In 24 patients of this group, radicality of resection consistent with S1 was achieved in the primary surgery, S2 radicality in 6 patients and S3 in the remaining two. The recurrence rate in patients with grade III meningioma (*n* = 8) was high, reaching 62.5% (5 patients), despite the resection being complete (S1) in all these cases.

Of the 38 conservatively treated patients, 16 died during our follow-up due to the age and other comorbidities. The remaining 22 cases were small incidental meningiomas, which we continue to monitor every 24 months and which remain without growth progression.

Forty-five patients were referred for radiosurgery using the Leksell gamma knife. The age range of the group was 22–67 years; the median was 35 years.

## Discussion

Meningiomas are tumours of a mostly benign nature with extraaxial growth, which, due to their biological behaviour, typically do not directly threaten the patient. The morbidity and mortality of benign forms of meningiomas can be influenced by other factors, mostly the tumour location and the radicality of resection, age and comorbidities [8, 15, 16]. Some authors have proven specific female-associated factors that increase the incidence of meningiomas in female population, which we confirmed also in our cohort [17, 18]. Also, several genetic changes could play an important role in meningioma pathogenesis [19]. Dumanski and Seizinger have proven several chromosome 22 aberrations [20, 21]. However, this knowledge did not lead to any change in therapeutic approach so far.

Meningiomas as benign, slow-growing lesions are not suitable for chemotherapy (as chemotherapy is generally more effective in fast-growing tumours). Atypical or anaplastic meningiomas (approximately 10% of cases), the biological behaviour of which is very similar to that of malignant glial tumours, are exceptions to this rule. Moreover, Palma et al. described a similar Kaplan–Meier curves in grade 3 meningiomas and anaplastic astrocytomas (grade 3), which confirms the above statement [22]. The first-choice drug in the chemotherapy of meningiomas is hydroxyurea followed by dacarbazine and adriamycin [15]. Grade 3 meningiomas belong to the most malignant forms of CNS tumours; fortunately, they are very rare—in our patient group, they constituted approx. 1% of all patients (i.e. 8 patients over 15 years). In all cases, the surgery has been followed by the combination of radiotherapy (54 Gy) combined with the administration of hydroxyurea, which has been consisted with recommendations of National Comprehensive Cancer Network [8, 16].

The pathophysiology of intracranial hypertension in slow-growing tumours differs from the sudden intracranial hypertension following, for example, epidural hematoma [16]. The rapid decompression caused by the removal of the tumour mass can also lead to a sudden shift of chronically dislocated structures and, in effect, to the damage of blood–brain barrier. In our cohort, 97 patients suffered from an acceleration of brain oedema after the surgery; on the other hand, only 17 patients required an additional surgical procedure—decompressive craniectomy. The combination of corticosteroids and 20% mannitol seems to be an appropriate treatment method in early postoperative period [23, 24]. A decision of performing a decompressive craniectomy should be considered wisely. There was no significant difference in the treatment outcome in our research group when comparing the patients with an accelerated brain oedema after the first surgery treated conservatively and surgically (decompressive craniectomy). However, two surgeries in a short period are quite risky due to the additional higher occurrence of infectious complications.

Generally, the meningioma management at the Brno University Hospital is guided by several factors. Factors that favour surgical treatment include the intracranial hypertension syndrome, age < 70 years, general condition of the patient, location of the tumour in the surgically suitable area, compression of important surrounding structures (vessels, cranial nerves) and tumour size over 2 cm [24]. If the anatomical situation does not allow S1 resection (cavernous sinus, chiasm, cerebellopontine angle) or if the patient suffers from grade III meningiomas and, last but not least, in the case of tumour recurrence surgery, adjuvant radiotherapy or (in benign variants) radiosurgery is considered.

Radiosurgery is also considered in cases of growing residual tumours [9]. The treatment with the Leksell gamma knife (which is the only radiosurgery technique used at our department) is preferred for smaller growing meningiomas located in the area of the chiasm (suprasellar, at a distance of at least 2 mm from the optic nerve), the cavernous sinus or the pontocerebellar angle. Older patients with multiple comorbidities, which have small growing meningiomas, are also suitable for stereotactic radiosurgery. The indication for radiosurgery is in each individual case consulted with a specialist; the decision procedure is guided by precise indication criteria taking into account the size of the tumour, the distance from the cranial nerves and/or from the brainstem [25, 26].

However, even radiosurgery may come with complications, such as postradiation oedema, necrosis and arachnoid scarring in the affected area. In our group of 45 radiosurgery-treated patients, one patient developed brain oedema, which did not react to conservative treatment and induced multiple seizures. Eventually, the patient’s condition necessitated surgical removal of the meningioma despite its small size (approx. 2 cm), which resolved the condition.

Tumour observation, i.e. “watch and wait”, is another valid strategy. It consists of regular MR and regular controls. We use it especially for asymptomatic (incidental) meningiomas in patients older than 70 years with numerous internal comorbidities. If tumour size progresses, we consider a radiosurgical procedure; surgery is the last resort [27, 28].

**Fig. 5:**
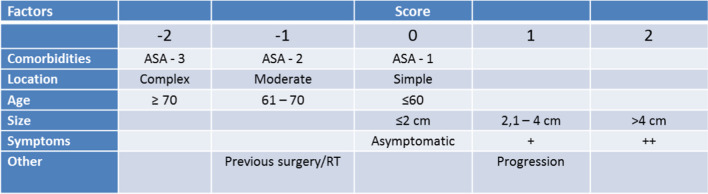
The table of CLASS score (ASA American Society of Anaesthesiologists, RT radiotherapy). The patients with the negative value should be considered for conservative treatment; on the other hand, if the patient achieves positive values, the surgery could be performed with a relative low risk of perioperative morbidity/mortality [29, 30]

**Fig. 6:**
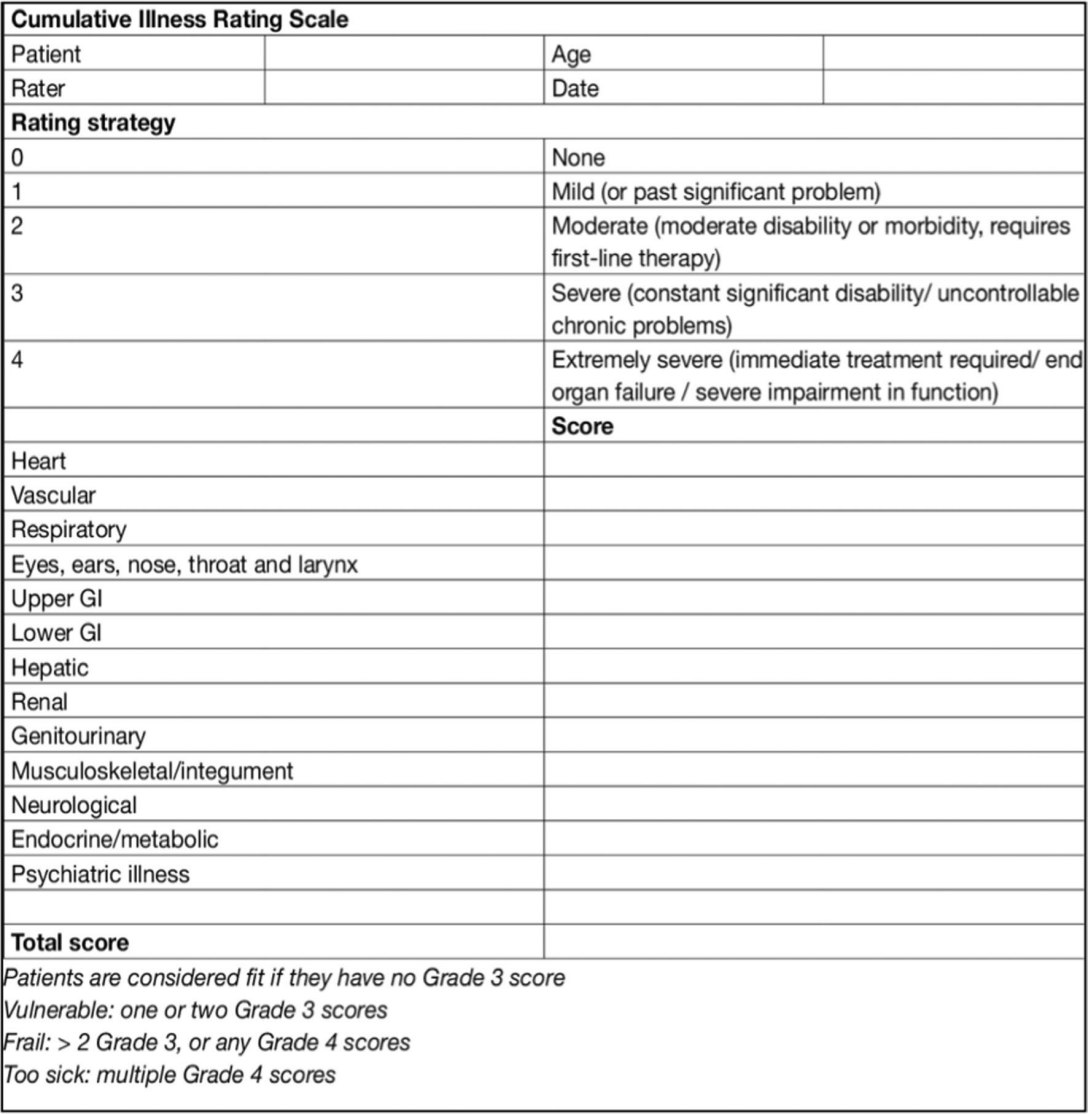
Geriatric Scoring Scale; deals with the pre-operative evaluation of the internal state of patients (GI gastrointestinal tract)

Multiple scoring systems can be used in decision on the method of treatment (surgical, radiotherapy, watch-and-wait). Among the most popular, CLASS score [29, 30], SKALE score [29, 31] or the Geriatric Scoring Scale [32, 33] should be named (Fig. 5). At our department, we use all these three systems, of which the CLASS score appears to be the most suitable in our experience (Fig.6). Patients with negative values should be considered for conservative treatment. If the tumour is growing—a radiosurgical procedure is indicated. In the cases with no progression observed on another MR scan performed after 6 months, we prefer “watch and wait” strategy. Patients with a positive value of CLASS score are suitable for the surgery with a low risk of perioperative morbidity.

The follow-up after the surgical procedure starts 3 months after the surgery with anew MRI scan and clinical examination. The frequency of regular controls is determined by the radicality of the resection and the histological type of the tumour. Grade I meningiomas after S1 and S2 resections are followed-up by MRI at 6, 12 and 24 months after the surgery. Subsequently, if the MRI is without any recurrence, further follow-ups are every 5 years. The overall progression-free interval in this subgroup was 6 years ± 7 months; from this perspective, we assume that higher frequency of regular MR scans is unnecessary. The approach we use is very similar to that recommended by Goldbrunner et al. [34]. It is necessary to point out that besides the aforementioned follow-ups at the neurosurgery department, the patients are still in the standard care of neurologists, who can refer them for another MR scan if clinical problems occur and then, the patient is sent to the neurosurgeon to further consideration. If the progression is just by a few millimetres, radiosurgery is indicated as a first-line therapy, after which the first MR scan is performed in 12 months. The MRI at that time point, however, may, paradoxically, show a pseudoprogression—signs of apparent tumour growth caused by the process of tumour necrosis. This, however, must not be mistaken for true progression and lead to further therapeutic intervention. Differentiation between these two can be made on the basis of gadolinium enhancement—in pseudoprogression, the contrast agent is not present. Another MR scan is performed 36 months after the radiosurgery in accordance with the guidelines [34]. In our group, no patient with grade I meningioma treated with S1 or S2 radicality suffered from another recurrence after the radiosurgery.

New surgery due to the recurrence of the meningioma is generally recommended in younger patients without comorbidities with, albeit slowly, increasing expansions (even though clinical manifestations may not have been present yet) [35].

Follow-ups after S3 and S4 resections of grade I tumours begin, similarly to the S1 and S2 groups, with MR scans after 6 and 12 months; the subsequent follow-up period is, however, shorter, every 12 months. If residuum progression was detected, the patients were referred for radiosurgery with MRI follow-ups every 12 months.

Patients with grade 2 meningiomas also undergo MRI after 6 and 12 months and then repeatedly every year (S1, S2) or every 6 months (S3, S4). In case of tumour recurrence, (providing the patient meets the indication criteria), the patient undergoes a new surgery, followed by radiotherapy and regular MR scans every 3 months. This is in accordance with recommendations by Kim [36]. Grade 3 meningiomas automatically proceed to adjuvant radiotherapy after the primary surgery and are subject to regular MRI scans every 6 months, regardless of the radicality of the resection. Regular whole-body PET follow-ups are also recommended in these patients due to the high risk (24%) of systemic metastases [37]. In our cohort, grade III meningioma occurred in 8 patients of which one patient died of residual progression within 12 months after the surgery, another underwent tumour recurrence surgery and was referred for subsequent radiotherapy, and the remaining 6 patients are in remission and in good clinical condition. It should be, however, noted that malignant forms of meningioma are very rare (1% of our cohort), which is similar to results reported elsewhere [38]. In conclusion, we should treat the malignant forms of meningioma in a way similar to the other glial malignant tumours [38]. Younis as well as Goyal proved in their studies that aggressive and immediate adjuvant therapy could improve the progression-free interval [38, 39]. This approach is also applied in our department.

## Conclusions

In conclusion, meningioma management in our department involves a multidisciplinary approach with advanced surgical and radiation therapy techniques to provide the best possible outcomes for patients. The availability of modern equipment and highly trained personnel in specialized centre ensures that patients receive timely and appropriate care. We proved that the treatment success is obviously associated with the radicality of resection [40]. Considering the benign types of meningioma (WHO I), we can say that the lower the radicality, the more likely the tumour is to recur [41]. Age and comorbidities could be other independent predictors of higher morbidity and mortality. Nevertheless, surgery may also be associated with complications. Therefore, finding the balance between a short-term morbidity due to a surgery and a long-term possible neurological deterioration following the “watch and wait” strategy remains one of the key issues of the therapeutic approach in the patients with meningioma [42]. This dilemma may be especially challenging in asymptomatic younger patients [43]. Finally, this comprehensive work could serve as a certain type of guidance in meningioma management, especially for smaller low flow neurosurgery departments due to the relatively large group of evaluated patients with a high complete removal rate and low 30-day morbidity/mortality ratio after the surgery. The second reason could be a relative low number of the patients with a need to another surgery due to the tumour recurrence in follow-up period. Most of the proved recurrences were treated with a stereotactic radiosurgery. The reason could be a relative short interval between the regular MR controls, so that the progression could be caught very early.

## Data Availability

Hereby I confirm, that all data, that support the findings are openly are available in FN Brno, Department of Neurosurgery.
